# A lectin-coupled porous silicon-based biosensor: label-free optical detection of bacteria in a real-time mode

**DOI:** 10.1038/s41598-020-72457-x

**Published:** 2020-09-29

**Authors:** Mona Yaghoubi, Fereshteh Rahimi, Babak Negahdari, Ali Hossein Rezayan, Azizollah Shafiekhani

**Affiliations:** 1 Division of Nanobiotechnoloy, Department of Life Science Engineering, Faculty of New Sciences and Technologies, University of Tehran, Tehran, Iran; 2 Department of Medical Biotechnology, School of Advanced Technologies in Medicine, Tehran University of Medical Sciences, Tehran, Iran; 3 School of Physics, Institute for Research in Fundamental Sciences, Tehran, Iran

**Keywords:** Analytical biochemistry, Biosensors, Optical sensors

## Abstract

Accuracy and speed of detection, along with technical and instrumental simplicity, are indispensable for the bacterial detection methods. Porous silicon (PSi) has unique optical and chemical properties which makes it a good candidate for biosensing applications. On the other hand, lectins have specific carbohydrate-binding properties and are inexpensive compared to popular antibodies. We propose a lectin-conjugated PSi-based biosensor for label-free and real-time detection of *Escherichia coli (E. coli)* and *Staphylococcus aureus* (*S. aureus*) by reflectometric interference Fourier transform spectroscopy (RIFTS). We modified meso-PSiO_2_ (10–40 nm pore diameter) with three lectins of ConA (Concanavalin A), WGA (Wheat Germ Agglutinin), and UEA (Ulex europaeus agglutinin) with various carbohydrate specificities, as bioreceptor. The results showed that ConA and WGA have the highest binding affinity for *E. coli* and *S. aureus* respectively and hence can effectively detect them. This was confirmed by 6.8% and 7.8% decrease in peak amplitude of fast Fourier transform (FFT) spectra (at 10^5^ cells mL^−1^ concentration). A limit of detection (LOD) of about 10^3^ cells mL^−1^ and a linear response range of 10^3^ to 10^5^ cells mL^−1^ were observed for both ConA-*E. coli* and WGA-*S. aureus* interaction platforms that are comparable to the other reports in the literature. Dissimilar response patterns among lectins can be attributed to the different bacterial cell wall structures. Further assessments were carried out by applying the biosensor for the detection of *Klebsiella aerogenes* and *Bacillus subtilis* bacteria. The overall obtained results reinforced the conjecture that the WGA and ConA have a stronger interaction with Gram-positive and Gram-negative bacteria, respectively. Therefore, it seems that specific lectins can be suggested for bacterial Gram-typing or even serotyping. These observations were confirmed by the principal component analysis (PCA) model.

## Introduction

According to World Health Organization (WHO) reports, dangerous water and food-borne diseases such as cholera, typhoid, dysentery, hepatitis, etc. are responsible for an estimated 2 million diarrheal deaths each year^1^. Therefore, providing inexpensive methods that can be run in the shortest possible time is a growing need for timely prevention of health crises. The conventional methods for microorganism detection (such as culture-, nucleic acid-, and immunological-based methods) mostly suffer from slowness, methodological complexity, and high cost that make them inefficient for rapid and real-time detection of pathogenic microorganisms^2^. Therefore, Over the past decades, categories of biosensors such as optical, electrochemical, and piezoelectric have attracted immense attention for diverse sensing applications^3^. Most optical biosensors have high sensitivity and high specificity. Their operation is cost-effective due to low material consumption. Furthermore, the real-time operation makes them fast and label-free monitoring makes them easy to operate. Therefore, they have gained full attention and so rapid progress^3–5^.

Along with the common optical approaches of fluorescence and surface plasmon resonance (SPR)^6^, recently, biosensors based on RIFTS such as PSi-based biosensors have opened new windows of opportunities for the design and fabrication of more effective analytical devices^7^*.* In addition to ease of fabrication, biocompatibility, and biodegradation^8^, PSi has outstanding features that make it an excellent choice as a transducer for biosensing. Its large surface area and a lot of hydride and hydroxyl surface groups allow for easy surface modification with a wide range of chemical methods^9^. Moreover, its solid substrate makes it a potentially regenerable and reusable biosensor. Based on these facts, in recent years, many PSi-based platforms are proposed for various applications such as drug delivery, characterizing of cellular processes such as phospholipid bilayer formation and discrimination of single nucleotide changes in DNA^10–12^. Detection of bacteria by this nanostructure (PSi) is another area of research with a large number of publications in the literature^13,14^.

Based on these facts, in recent years, many PSi-based platforms are proposed for drug delivery, characterizing of cellular processes such as phospholipid bilayer formation and discrimination of single nucleotide changes in DNA and bioassay^10–13^. Detection of bacteria by this nanostructure (PSi) is another area of research that has been accompanied by the publication of a considerable amount of literature. Examples in this area are the following researches:

An antibody conjugated nanopore array based on PSi has been introduced for the immunosensing of *E. coli* by RIFTS^15^. As well as regarding the size-exclusion filtering capabilities of PSi structure, it could be used as both a filter and a sensor for the detection of target molecules from complex biological samples^16^. Also, a porous SiO_2_/hydrogel hybrids structure immobilized with the antibody via a biotin-streptavidin system is constructed for label-free detection of bacterial by RIFTS. Recently a photonic crystal of porous silicon has been employed for bacterial detection based antibody-antigen interaction. In addition to one-dimension photonic crystal configurations of PSi, other sophisticated structures such as Bragg mirrors and microcavities have been employed for biological application^14,17–19^.

Up to now, the antibodies and nucleic acid-based probes have been the most used bioreceptors in biosensors design. But their use has been associated with the challenges. For example, antibodies, despite their unique specificity and selectivity, are unstable and expensive. In fact, glycan moieties are poor immunogen and for this reason, the production of their high specific antibodies is very hard^20^. The nucleic acid-based probes such as aptamers also suffer from long, labor-intensive, and costly production processes. Moreover, the target structure of antibodies and aptamers must be well-defined. Otherwise, the cross-reactivity probability increases and undermines the accuracy of the results^21,22^. Thus in recent years, lectins have been opened up broad prospects for the application in biosensing studies. The lectins are a group of proteins with plant, microbial or animal origin. They recognize and bind to specific glycan moieties of glycoproteins or glycolipids^23^.

In contrast to antibodies, lectins are inexpensive and more stable. They have a smaller size that can provide an appropriate surface density. Also, the specificity of lectins is based on the presence of certain carbohydrate residues in glycan moieties and is independent of a particular sequence. But their major drawback is that they have less specificity than antibodies. Although, due to their potential for polyvalent interaction (mostly having more than one binding site) with carbohydrates, this limitation is improved, and results in an affinity comparable to immunological interaction^24^*.*

Although there is a growing interest in the design of lectin based biosensors, the main focus of this researches has been on the analytical label-free methods such as SPR, quartz crystal microbalance (QCM), and electrochemical impedance spectroscopy (EIS)^23,25^. To the best of our knowledge, there is only one study on the use of lectin-modified PSi to evaluate antimicrobial susceptibility by the RIFTS method without focusing on the role of lectin^26^. The aim of this research has therefore been to investigate the use of lectins instead of antibodies to modify the surface of PSi for label-free detection of bacteria by the RIFTS method. In this work, we sought to detect *E. coli* and *S. aureus* by three types of lectins (ConA, WGA, and UEA) as bioreceptors and compared our results with other investigations using antibodies as biorecognition elements.

## Material and methods

### Fabrication of PSi

Highly boron-doped (p-type) silicon wafers (0.001–0.009 Ω cm resistivity,  ⟨100⟩-oriented) were purchased from Latech Scientific Supply, Singapore. The wafers were cut into 8 mm × 8 mm squares pieces by laser cutting. At first, silicon samples were washed by soap and deionized (DI) water. Then, sonicated in methanol and ethanol mixture (1:1 volume ratio) for 5 min and rinsed with DI water. The etching process carried out in a Teflon cell by using an electrolyte solution consisted of HF (39%) and absolute ethanol (3:1 volume ratio). Due to the formation of a parasitic layer (with a much smaller pore size than the desired size) on top of the surface, a two-step protocol was followed to eliminate this layer^10,27^. First, the silicon pieces were subjected to anodic etching under a constant current density of 100 mA cm^−2^ for 30 s and then were rinsed with methanol and were dried under a nitrogen stream. This initial layer was destroyed by sonication in an ethanolic solution of NaOH (1 M) (4 g NaOH + 10 mL EtOH + 90 mL DI water) for 3 min. Then samples were washed three times with abundant ethanol. In the second step, the main sensing layer was prepared by the etching of previous substrates in the formerly mentioned concentration of electrolyte and at a constant current density of 130 mA cm^−2^ for 60 s. After etching, the PSi samples were rinsed with methanol and were dried under a nitrogen stream.

### PSi surface modification

**Figure 1:**
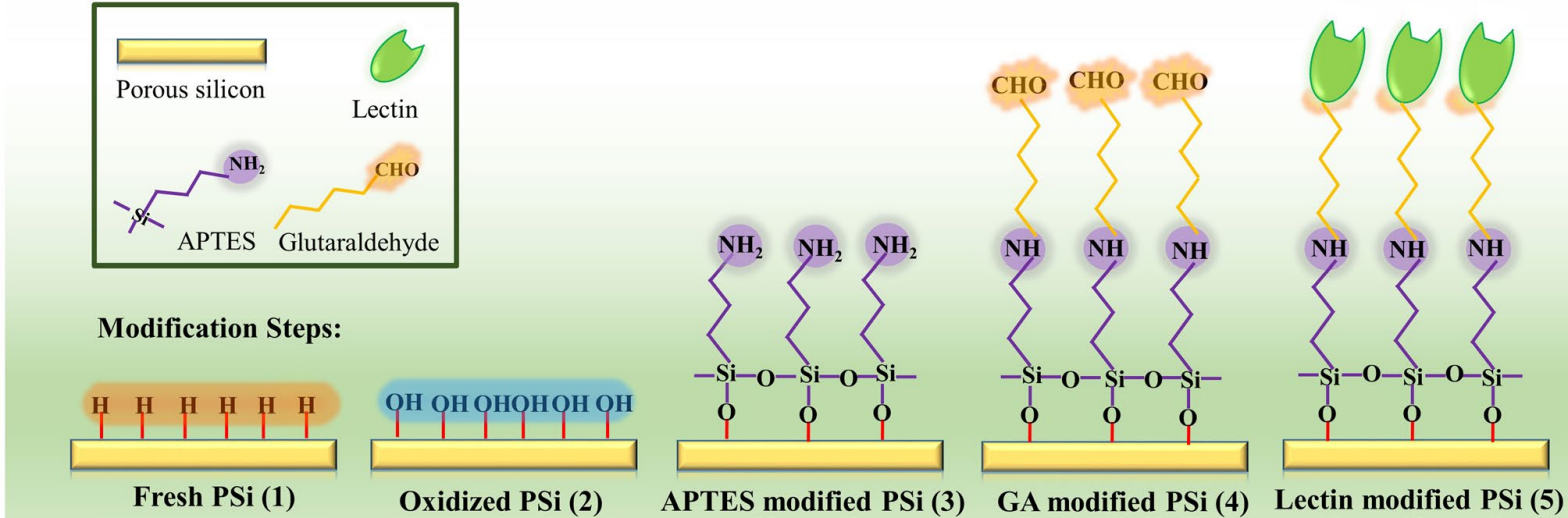
Surface modification of PSi as transducer upon each modification step.

Freshly-etched PSi samples were passivated by chemically oxidization in hydrogen peroxide (35%, v/v; Merck, Germany) for different time (1.5, 4, or 24 h) in ambient and dark condition. For some samples, a thermal pretreatment in an oven at 65 ºC for 1 h was applied. Best oxidation conditions (analyzed by Fourier transform infrared spectroscopy, FTIR; Fig. 4A) were chosen to continue the modification process. Best oxidized PSi samples were dipped into ethanolic APTES solution (10%) for 1 h to activate the surface and then were rinsed with absolute ethanol and dried in air. They subsequently were incubated in 2.5% GA solution in 20 mM HEPES buffer (pH = 7.4) for 1 h at room temperature. Finally, they were washed thrice with phosphate-buffered saline (PBS, pH = 7.4) to remove excess GA. For lectin immobilization, GA-modified substrates were dipped into 1 mL of 2 μM concentration of ConA (from *Canavalia ensiformis*), WGA (from *Triticum vulgari*s) or UEA (from *Ulex europaeus*) lectins in PBS for 1 h at 37 ºC. In these circumstances, the aldehyde groups of GA react with the amino groups of lectins (protein) leading to amide bond formation. After that, the substrates were washed thrice with PBS to remove unattached lectin molecules. All of the surface modification steps are schematically displayed in Fig. 1.

### Bacterial culture

*Escherichia coli* (ATCC 35218) and *S. aureus* (ATCC 29213), *Klebsiella aerogenes* (ATCC 13048), and *Bacillus subtilis* (ATCC 6051) strains were purchased from the Pasteur Institute microbial collection of Iran. The bacteria strains were cultivated in a nutrient broth medium (50 mL) and incubated at 37 °C with shaking for 24 h. After the incubation period, 10 mL of the overnight culture was centrifuged at 6,000 rpm for 10 min. The supernatant was discarded and bacteria biomass resuspended in saline solution. The bacterial suspension was used for the preparation of serial dilution (10^–1^ to 10^–7^) with saline. The 10^–3^ to 10^–5^ dilutions were used for the counting of viable bacteria cells by the drop-plate method and biosensing experiment simultaneously. Also, we used the fresh overnight *E. coli* culture medium as a complex sample. The serial dilutions (10^–3^ to 10^–5^) of overnight culture medium were prepared and straightly applied for biosensing experiment and plate counting.

### PSi layer characterization

The structure, pore size, and surface morphology of the PSi samples were characterized by field emission scanning electron microscopy (FE-SEM; MIRA3, TESCAN, Czech Republic) working at 15 kV acceleration voltage of the electron gun. Pores diameter distribution was obtained by ImageJ software (ImageJ; National Institutes of Health, Bethesda, MD, USA). The chemical compositions of the surface were investigated with FTIR (Tensor 27, Bruker, USA) in ATR (attenuated total reflectance) mode. All the spectra were obtained from an average over 16 scans with a resolution of 4 cm^−1^ in the wavenumber range 400–4,000 cm^−1^. To evaluate the wettability of samples, the contact angle (CA) between the surface and DI water droplet (4µL) was measured by a CA system (CA-500A, SharifSolar, Iran). CA measurements at five different points of the substrate surface were carried out and the average of them was reported.

**Figure2:**
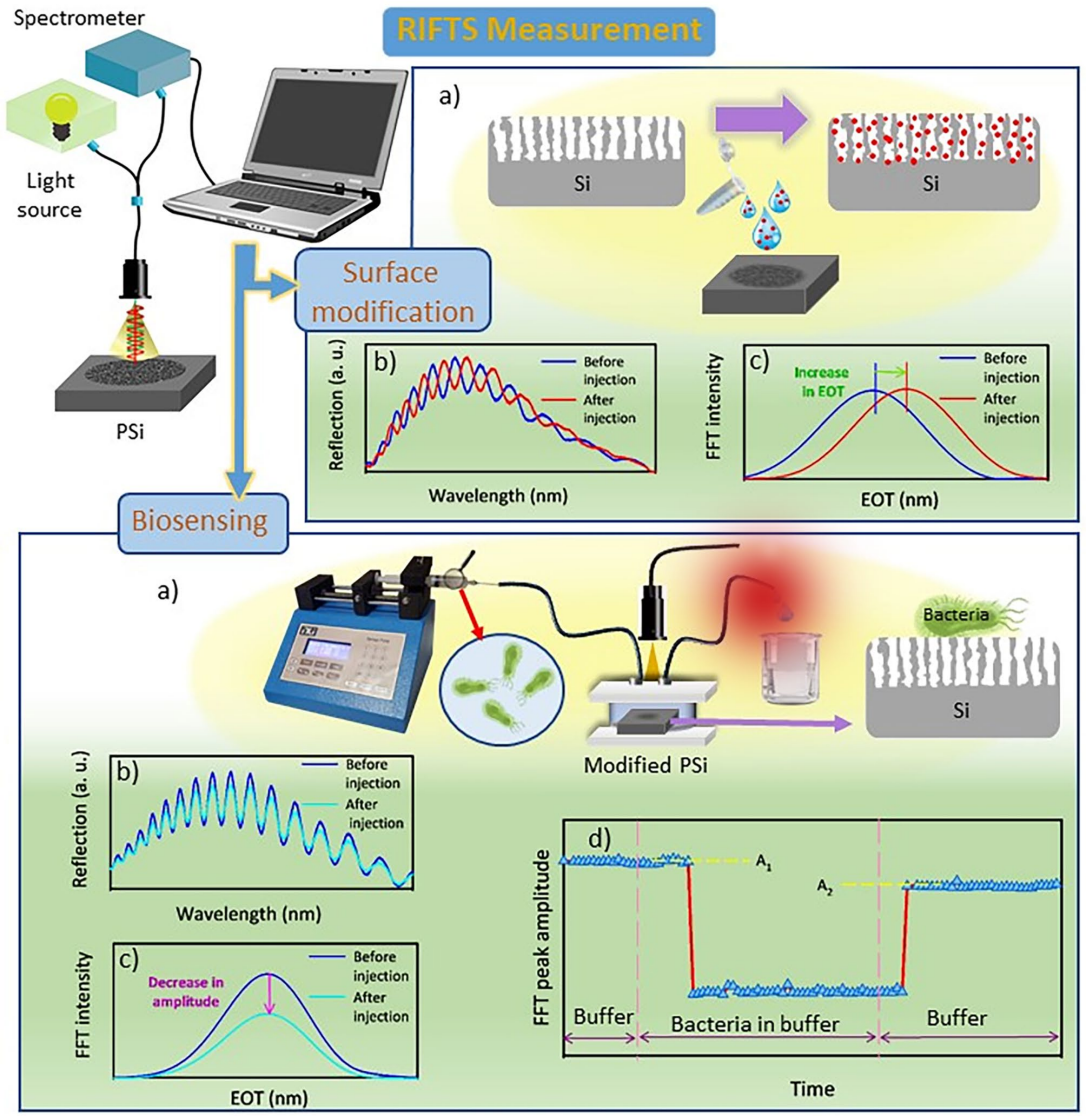
Schematic of RIFTS measurement. Surface modification box: (**a**) the penetration of analyte molecules in pores or change the surface chemistry during surface modification process which alters EOT, (**b**) reflection spectra from PSi in each step in part a and (**c**) the FFT intensity extracted from the spectra in part (**b**). Biosensing box: (**a**) biosensing monitoring of bacterial suspension by a handmade fluidic system, (**b**) reflection spectra from PSi in two steps in part (**a**); (**c**) the FFT intensity extracted from the spectra in part (**b**); (**d**) Real-Time monitoring of biosensor response extracted from part c during the initial washing, bacterial expose and final washing steps.

For RIFTS measurements, a bifurcated optical fiber was used to focus the white light from a tungsten-halogen light source on the sample surface and transfer the specularly reflected beam to the spectrometer (USB4000, Ocean Optics, USA) as illustrated schematically in Fig. 2, main part. The reflected spectra were recorded in the range of 400 to 1,000 nm at a spectral resolution of 0.5 nm with a spectral acquisition time of 10 ms. For surface modification study by RIFTS measurements, in each modification step (Fig. 2, surface modification box, part a), the reflected spectrum (Fig. 2, surface modification box, part b) was transformed by fast Fourier transformation (FFT) which was computed by the IGOR software (Wavemetrics Inc., Oregon, USA). This transformation yielded a peak located at the value of effective optical thickness (EOT, twice the product of the refractive index and thickness values of the porous layer^10^; Fig. 2, surface modification box, part c). The porosity, refractive index, and thickness of the porous layer were determined by the method similar to the surface modification box in Fig. 2 and using spectroscopic liquid infiltration method (SLIM)^28^. It means that the reflectance spectra were obtained from the surface of fresh-PSi in two different media (air and methanol) and corresponding FFT values were calculated. By applying the SLIM algorithm of IGOR PRO software to these values, porosity, refractive index, and thickness of the porous layer were calculated.

For biosensing measurements, modified PSi samples were fixed on a handmade fluidic system (Fig. 2, biosensing box, part a). This includes a fixed plexiglass well connecting to a syringe pump with an inlet tube for injection and an outlet tube for the evacuation of liquids (saline buffer and bacterial solution). In each experiment, an initial washing step was performed by passing saline buffer for 10–15 min over the sample at a flow rate of 200 µL/min. In the second step, bacterial suspension in saline (in dilutions of 10^–3^ to 10^–5^ cells mL^−1^) was injected with the previous flow rate (200 µL/min) and the surface was allowed to incubate with it for 25–30 min at ambient temperature. Finally, the surface was rinsed with saline again for 10–15 min at a flow rate of 50 µL/min. During all of the foregoing processes, the reflective spectrum was recorded every 2 min. Since the bacteria size is several orders of magnitude larger than the pore diameters of the porous layer, adsorption of bacteria occurred only on top of the pores of PSi. It changes the contrast between the refractive index of PSi and the refractive index of ambient. Consequently, the amount of reflected light (Fig. 2, biosensing box, part b) and the peak amplitude of its FFT values (Fig. 2, biosensing box, part c) were decreased in harmony with other research^13,29^. After the subsequent washing step and with the removal of unbounded species, the intensity of the reflection spectrum was increased again. However, depending on the sensitivity of the sensor, this value remains lower than the base signal. Real-time monitoring of the biosensing process was carried out, by extracting the FFT peak amplitude vs. time (Fig. 2, biosensing box, part d). Finally, the FFT peak amplitude change in percentage for PSi lectin-modified biosensor was calculated by the following equation:

1 $$FFT\;peak\;amplitude\;change\;(\%)=\frac{A_{1} -A_{2}}{A_{1}} \times 100$$

where A_1_ and A_2_ are the average FFT peak amplitude before and after exposure to the bacteria (Fig. 2, biosensing box, part d). All errors, which are reported in this study are the standard deviations.

### Principal component analysis (PCA)

Principal component analysis is a multivariate projection method designed to extract and display the systematic variation in a data matrix. The starting point for PCA is a matrix of data with N rows (observations) and K columns (variables)^30^. In our case, elements of the data matrix were the FFT peak amplitude changes (responses of biosensors). The columns representing the responses of a specific lectin to different bacteria at different concentrations. However, the rows representing the responses to a distinct bacteria at a definite concentration from different lectins. Principal component analysis (PCA) was applied to this matrix and scores plot and loading plot were obtained in Sigmaplot (Systat Software Inc.).

## Results and discussion

### Characterization of PSi by FE-SEM

**Figure 3:**
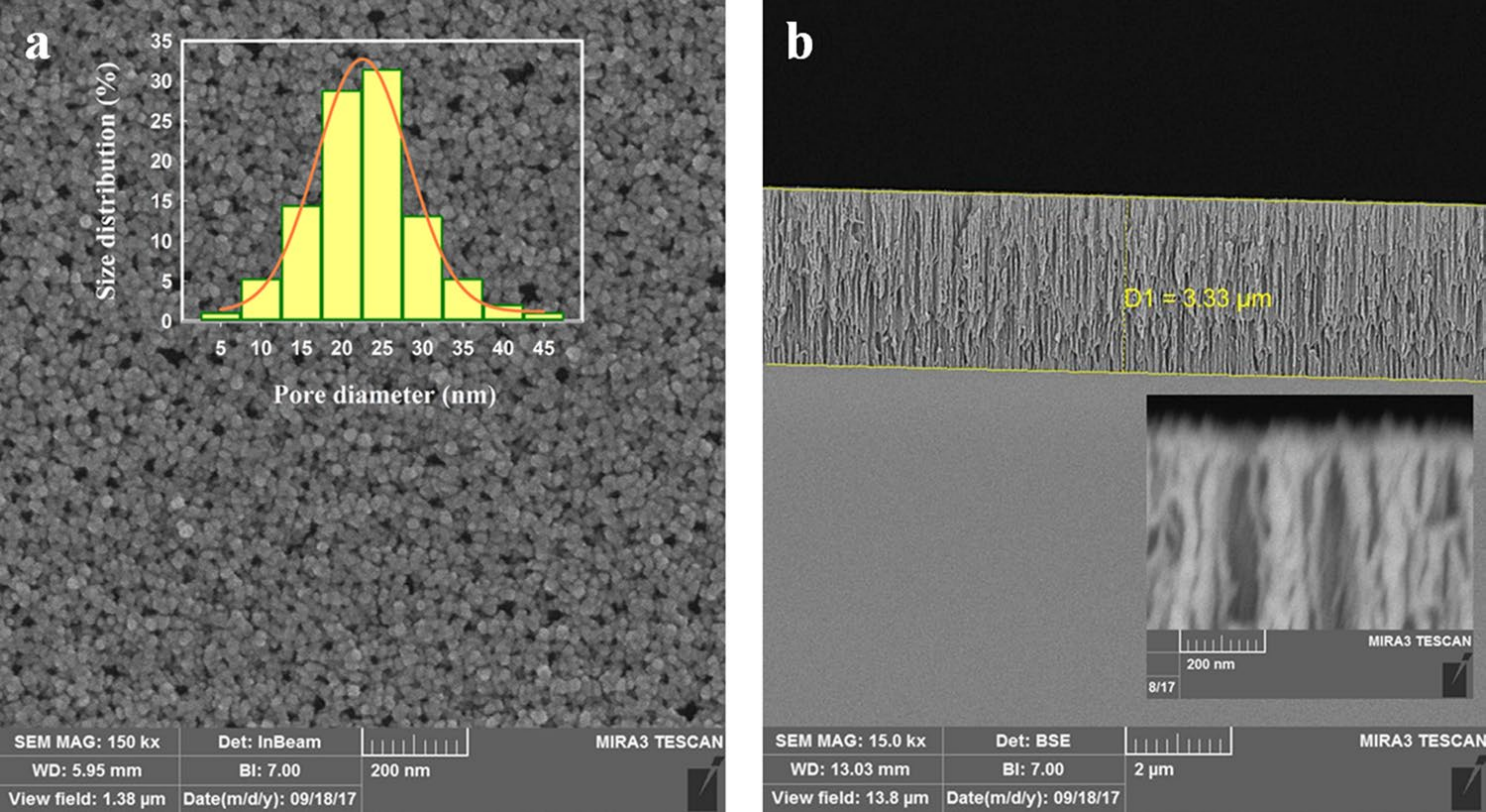
FE-SEM images of PSi: (**A**) top view (main plot) and pore diameter distribution (inset plot), (**B**) and cross-sectional view (inset: image with higher resolution).

The characteristics of the PSi layer i.e. thickness and pore size were studied by FE-SEM after oxidation. Figure 3A illustrates the pores on the surface and the inset plot shows the pore size distribution (5 to 45 nm) which the majority of pores have the size between 20 and 25 nm. The cross-section imagining of the porous layer reveals a thickness of about 3.3 µm (Fig. 3B) and the inset plot illustrates the cross-section image near the surface with higher resolution.

### PSi surface modification study

Freshly prepared PSi surface is rich in hydride groups (Si_x_SiH_y_)^9^ that are highly reactive, unstable, and inherently susceptible to oxidation or hydrolysis, even in environmental conditions. It changes the physicochemical and optical properties of the PSi layer over time^31^. For example, in biosensing purposes, these unwanted reactions increase the gradual dissolution of atoms from the surface and detach the biorecognition elements^32^. Additionally, it has been shown that hydride groups are strong reducing agents that make the surface incompatible to conjugate with many biological or non-biological materials^33^. Hence, surface passivation of PSi is a critical step to stable its surface for application at biological research fields^34^. Thus, the fresh PSi samples were modified as reported in the “PSi surface modification” section. To verify the accuracy of PSi layer modification, the surface chemical changes were monitored by FTIR, CA analysis, and RIFTS after each step.

**Figure 4:**
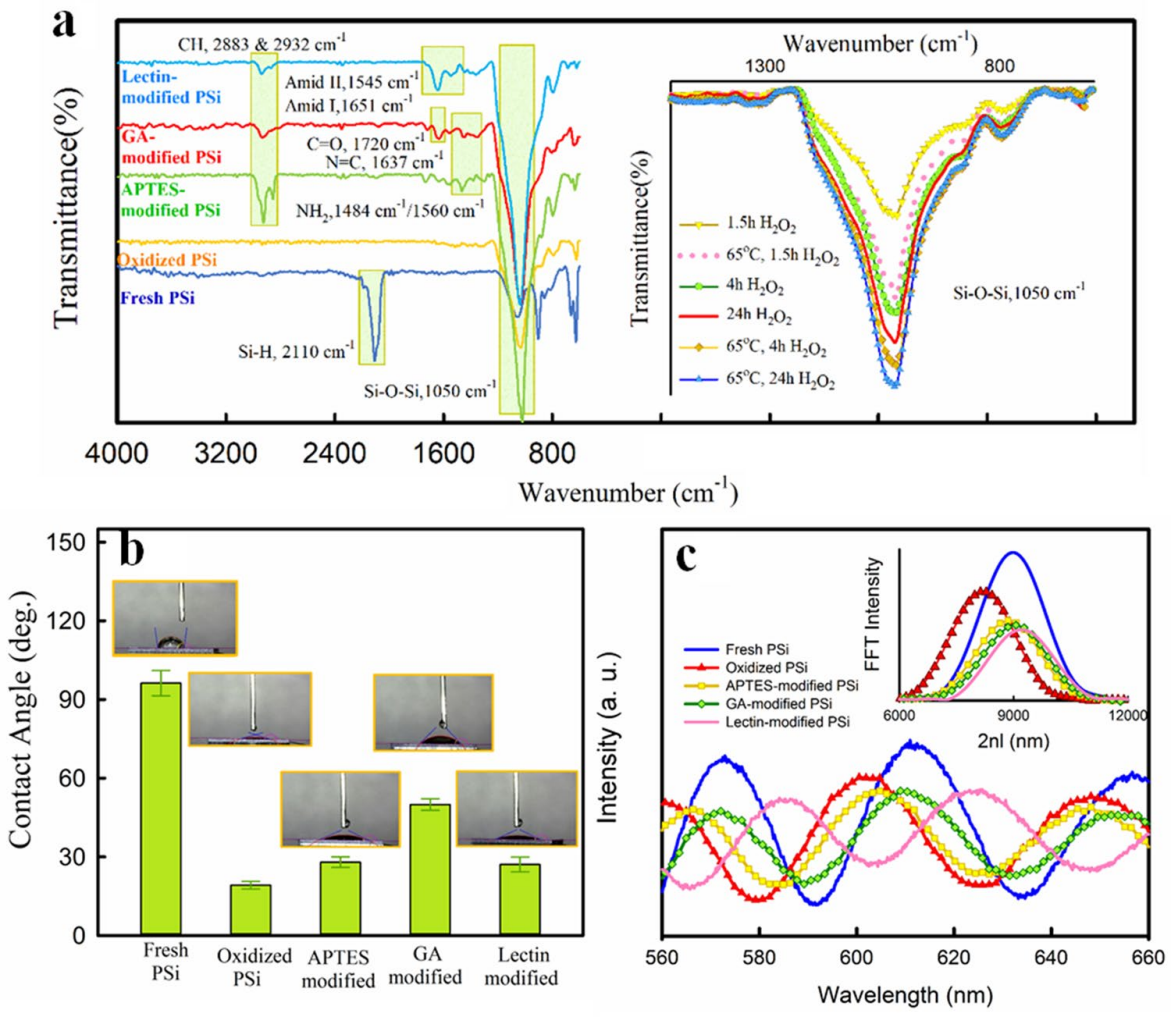
Step by step study of PSi surface modifications by (**A**) FTIR analysis (inset: comparison between different oxidation conditions on Si–O–Si bonds), (**B**) CA measurement, and (**C**) RIFTS method (inset: corresponding FFT values).

During the surface modification, the chemistry of the sample surface undergoes a considerable change that can be monitored by FTIR analysis (Fig. 4A). These results are similar to those reported by other^35,36^. After oxidation, the appearance of typical peaks attributes to Si–O–Si bonds at 1,000 to 1200 cm^−1^ and under 1,000 (attribute to O_n_SiH_x_ species) in FTIR spectra verify the successful oxidation process. Therefore, to achieve the best oxidation condition (among the six different oxidation processes) as mentioned in the “PSi surface modification” section, the Si–O–Si bond absorption peak was more accurately investigated. As Fig. 4A (inset) indicates, the sample oxidized by a thermal pretreatment in an oven at 65 °C for 1 h and subsequently immersion in H_2_O_2_ for 24 h presents a stronger absorption peak. Thus, this oxidation method has been selected as the final protocol for the oxidation of samples. After APTES modification ((Fig. 4A, main plot), the band vibrations mods of this molecule leave two distinct absorption regions in 2,800–3,000 cm^−1^ and 1,550–1,650 cm^−1^. The first one is assigned to the symmetric and asymmetric stretching modes of C–H from APTES backbone in 2,932 cm^−1^ and 2,883 cm^−1^ respectively. Another region is related to NH_2_ deformation modes of APTES amine groups at 1,484 cm^−1^ and 1,560 cm^−1^. After GA treatment, the spectrum depicts two peaks assigned to CH_2_ deformation and C=O (aldehyde group) stretching mode respectively at 1,407 and 1,720 cm^−1^. One another peak attributed to the N=C bond between the silane layer and GA appeared between 1,630 and 1,650 cm^−1^. Finally, the lectin immobilization step was verified by the appearance of two significant absorbance peaks attributed to amide I and amide II bands at 1,653 and 1,545 cm^−1^ respectively.

As already mentioned the surface of freshly prepared PSi due to the abundant hydride groups displays extremely hydrophobicity that limits it for the biomedical application taking place in aqueous-based medium^9^. However, after oxidation, as shown in Fig. 4B, a sharp decrease in CA from 96º  ± 13º to 19º  ± 8º validates the successful oxidation process. After APTES and GA treatment, we observed an increase in hydrophobicity (28º  ± 6º and 50º  ± 11º respectively) due to the carbon backbone of these molecules. But after the lectin immobilization, the presence of hydrophilic functional groups of the protein structure compensates for this decrease and CA decreased to 27º ± 4º. These results are in agreement with other reports indicating that changes in PSi surface chemistry during of modification process greatly affect the wettability behavior of it^10,37^.

Since the refractive index (and consequently EOT parameter) of the PSi layer is greatly affected by its surface chemistry, another way to evaluate the success of the modification process is the assessment of reflective spectra. Figure 4C (main plot) demonstrates these reflective spectra for the fresh PSi layer and after each modification step. FFT of these spectra has been illustrated in the inset of Fig. 4C. As it has been shown, upon oxidation a decrease in EOT was observed (8.89 ± 0.13 µm to 8.12 ± 0.14 µm). This is attributed to the formation of a thin SiO_2_ layer on pore surfaces that has a lower refractive index than Si skeleton^38^. In contrast, modification of the surface by APTES and GA molecules, increased EOT to 8.78 ± 0.14 µm and 8.95 ± 0.19 µm respectively, due to organo-silane layer formation that increases the effective refractive index of layer^37^. Furthermore, through the lectin modification, due to the amide bond formation, EOT increased more (9.16 ± 0.14 µm). The porosity and refractive index of the fresh PSi layer were calculated by the SLIM method. These parameters were 82 ± 5% and 1.5 ± 0.3 respectively averaged over three samples. Also, the SLIM method yielded a thickness of 3.5 ± 0.5 µm for the PSi layer which is in agreement with the FE-SEM result (3.3 µm).

### Evaluation of biosensor performance

**Figure 5:**
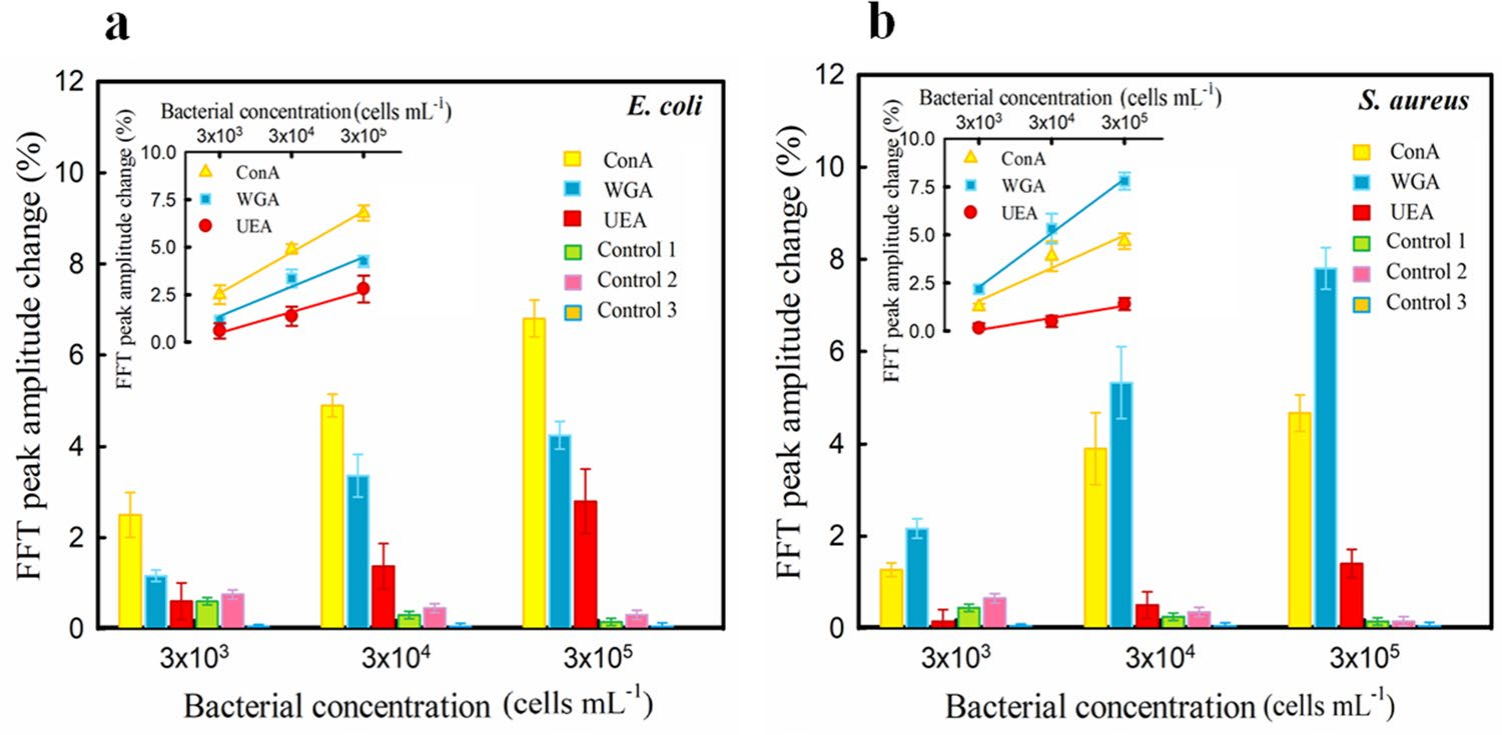
FFT peak amplitude change percentage of PSi modified by three different types of lectin (ConA, WGA, and UEA) at the presence of different concentrations of (**A**) *E. coli* and (**B**) *S. aureus*. Control tests: fresh PSi as control 1, GA-modified PSi (without lectin) as control 2 and DI water on ConA-modified PSi as control 3. (R-squared values: 0.9955 for *E. coli*-ConA, 0.9435 for *E. coli*-WGA, 0.9709 for *E. coli*-UEA, 0.9093 for *S. aureus*-ConA, 0.9950 for *S. aureus*-WGA, 0.9394 for *S. aureus*-UEA).

Carbohydrates are one of the four major groups of macromolecules that are found on the surface of bacterial cells such as all other living organisms. In addition to the nutritional role of carbohydrates as an energy resource, many of the vital functions of living cells such as movement, structural protection, cell–cell recognition, cell junction, carry out through proteins and lipids that are glycosylated^39^. Huge structural diversity at cell surface glycans in contrast to DNA and proteins (even between cells from one species) introduce them as a unique identity card for cell identification^40^. The similarity of physicochemical properties, despite the unique structural complexity of carbohydrate, limits the detection of them with common methods based on the chromatography or spectroscopy for biosensing purposes^24,41^. Nevertheless, lectins, relying on their ability to detect not only the composition of carbohydrates but also the configuration of bonds between the sugar residues, are the most effective tools in this area^42,43^. Therefore, in this study, lectin-carbohydrate interaction has been considered as the basis of detection. The function of this biosensor is evaluated and compared with three lectin types having different sugar-binding specificity to choose the best design with the highest affinity and performance. Figure 5 illustrates the response [FFT peak amplitude change percentage, Eq. (1)] of PSi modified by these three types of lectin (ConA, WGA, and UEA) to the three different concentrations (3 × 10^3^, 3 × 10^4^ and 3 × 10^5^ cells mL^−1^) of *E. coli* (Fig. 5A) and *S. aureus* (Fig. 5B). For comparison, the response of fresh PSi (control 1) and modified PSi (without lectin; control 2) to both types of bacteria in three different concentrations and ConA-modified PSi (control 3) to DI water were measured. The figure shows some interesting results which are summarized as follows:

- Each of the three types of modification for PSi (by ConA, WGA, and UEA) has responded to both types of bacteria (*E. coli* and *S. aureus*). These responses were considerable except modification by UEA at a bacterial concentration of 3 × 10^3^ cells mL^−1^. In this case, the responses were comparable to the responses of control tests. Table 1, compares the characteristics (LOD and linear range) of the presented biosensor with examples of other reports. It is apparent from this table that the obtained LOD values and linear ranges of our biosensors are comparable to similar quantities of other reports based on QCM, SPR, and etc. biosensors with lectin modification. Besides, the data in Table 1 shows no observable contrast between the performance of lectin-modified and antibody-modified PSi biosensors based on RIFTS. However, lectins have a much lower affinity than the antibodies (dissociation constant of 10^–4^–10^−5^ M for lectins vs. 10^−8^ M for antibodies)^44^, there are several possible explanations for our observations. This results could be attributed to the relatively smaller size of lectin molecules (in comparison with antibodies) which allows for the higher density of binding sites on the surface for these molecules to produce comparable sensitivity^45^. Polyvalency (multiple binding sites) of lectins may be another important factor that compensates for this limitation.
- As illustrated no significant change was observed in FFT peak amplitude for control tests. This observation indicates that the lectin-carbohydrate interaction is the key role to bacterial bonding in this experiment and the physical absorption of bacteria on the surface is much negligible to trigger a considerable change in the reflection spectrum.
- There was a linear relationship (in semi-logarithmic scale) between the FFT peak amplitude change percentage and bacterial concentration in the range of 3 × 10^3^ to 3 × 10^5^ cells mL^−1^ for each of the three types of modification and both type of bacteria (insets in Fig. 5A,B). As shown the values of R-squared are between 0.9093 to 0.9950. However, when the concentration of bacteria approached 10^6^ cells mL^−1^ for both cases, the FFT peak amplitude change percentage was no longer be linearly proportional to concentration due to the overcrowding bacteria cells adjacent to the PSi surface which increase light scattering. Also in concentrations less than 10^3^ cells mL^−1^, no distinct signals were observed (data not shown).
- The biosensor responses were different for two types of bacteria based on lectin types. PSi modified by ConA had the highest responses to *E. coli* in all three concentrations. However, modification by WGA was the best one to efficiently detect *S. aureus* in the same foregoing concentrations. In contrast, PSi modified by UEA showed the lowest responses to both types of bacteria in all measuring concentrations. It is suggested that these observations are caused by the different binding affinity between lectins and bacterial species. Such different affinity profiles can be evaluated from two perspectives: (1) Based on the binding affinity levels of used lectins and (2) Based on the structural difference between the two bacterial species. Based on the first perspective and as mentioned previously the affinity level of lectins greatly depends on their structure and number of the sugar-binding sites. The more the number of binding sites, the greater the binding affinity. WGA with eight binding sites which are complementary to 6 or 8 β-1,4-GlcNAc units^55^ in contrast to ConA with four binding sites for 4 α-d-mannosyl or α-d-glucosyl residues of sugar motifs^56^. However, UEA has only one binding site for α (1,2) linked fucose residues of oligosaccharide motifs^57^. It seems possible that the low responses of our UEA-modified biosensors are due to this factor. Without a doubt, the presence of sufficient amounts of the target molecule in addition to having multiple binding sites is another essential condition for the occurrence of this event. Based on the second perspective, it should be noted that Gram-positive and Gram-negative bacteria have different cell wall structures. The peptidoglycan (or murein) is the main cell wall component of both groups that like a rigid envelope surrounds the cytoplasmic membrane. This polymer contains sugar (glycan) chains of β-(1,4) linked N-acetylglucosamine and N-acetylmuramic acid, which are cross-linked by peptides bridges. The Gram-positive bacteria cell wall consists mainly of a thick peptidoglycan layer and many other secondary polymers as the outmost part of bacteria. Gram-negative bacteria have complicated cell wall that in which a thin peptidoglycan layer enclosed by an outer membrane rich in a macromolecule known as lipopolysaccharide (LPS) and other lipo- or glycoproteins. LPS molecule is made up of three distinctive domains includes of lipid A, core polysaccharide, and O-antigen. O-antigen is the outmost part and consists of unique repeating oligosaccharide subunits that vary even in strain level^58^. Besides, the peptidoglycan is the main content of the cell wall structure (up to 90%). Thus *N*-acetyl-d-glucosamine (target of WGA) containing motifs are one of the most abundant structures in the external decoration of Gram-positive bacteria. In Gram-negative bacteria, such a parallel role can be imagined for d-glucose and d-mannose containing motifs of O-antigen and other glycosylated proteins or polymers. Hence, given the availability of two above-mentioned conditions (The plurality of binding sites and the frequency of the target molecule), the obtained results are logically interpretable and acceptable.

**Table 1:** Review of the characteristics of some bacterial biosensor reported in the literature.

Assay principle	Target bacteria	Bioreceptor	LOD^*^ (cells·mL^−1^)	Linear range (cells·mL^−1^)	References
QCM	*C. jejuni*	Lectin (ConA)	103	10^3^–2 × 10^4^	^46^
SPR	*E. coli O157: H7*	Lectin (WGA)	3 × 10^5^	NA	^47^
EIS	*E. coli*	Lectin (ConA)	75	10^2^–10^5^	^48^
EIS	Sulfate reducing bacteria	Lectin (ConA)	1.8	1.8 × 10^0^–1.8 × 10^7^	^49^
Piezoelectric	*E. coli*	Lectin (ConA)	5 × 10^6^	5 × 10^6^–2 × 10^7^	^50^
ECL	*E. coli O157: H7*	Lectin (ConA)	127	5.0 × 10^2^–5.0 × 10^5^	^51^
Chemiresistiv	*E. coli K12, E. faecalis, S. mutans,S. typhiE. coli K12, E. faecalis, S. mutans,S. typhi*	Lectin (ConA)	4.7 × 10^3^, 25 cfu/mL, 7.4 × 10^4^, 6.3 × 10^2^	Var.	^41^
QCM	*E. coli*	Lectin (ConA)	7.5 × 10^2^	7.5 × 10^2^ –7.5 × 10^7^	^52^
RIFTS-PSi	*E. coli*	OAKs	103	10^3^–10^5^	^53^
RIFTS-PSi	*L. acidophilus*	Aptamer	106	–	^29^
RIFTS-PSi	Ammonia-oxidizing bacteria	DNA sequence	–	–	^14^
RIFTS-PSi	*E. coli*	Antibody	103	10^3^–10^7^	^15^
RIFTS-PSi	*E. coli*	Antibody	103	10^3^–10^5^	^13^
RIFTS-PSi	*E. coli K12*	Antibody	104	103	^54^
RIFTS-PSi	*E. coli*	Lectin (ConA)	103	3 × 10^3^–3 × 10^5^	This work
RIFTS-PSi	*S. aureus*	Lectin (WGA)	9 × 10^2^	3 × 10^3^–3 × 10^5^	This work

*LOD = 3.3 × standard deviation/slope.

**Figure 6:**
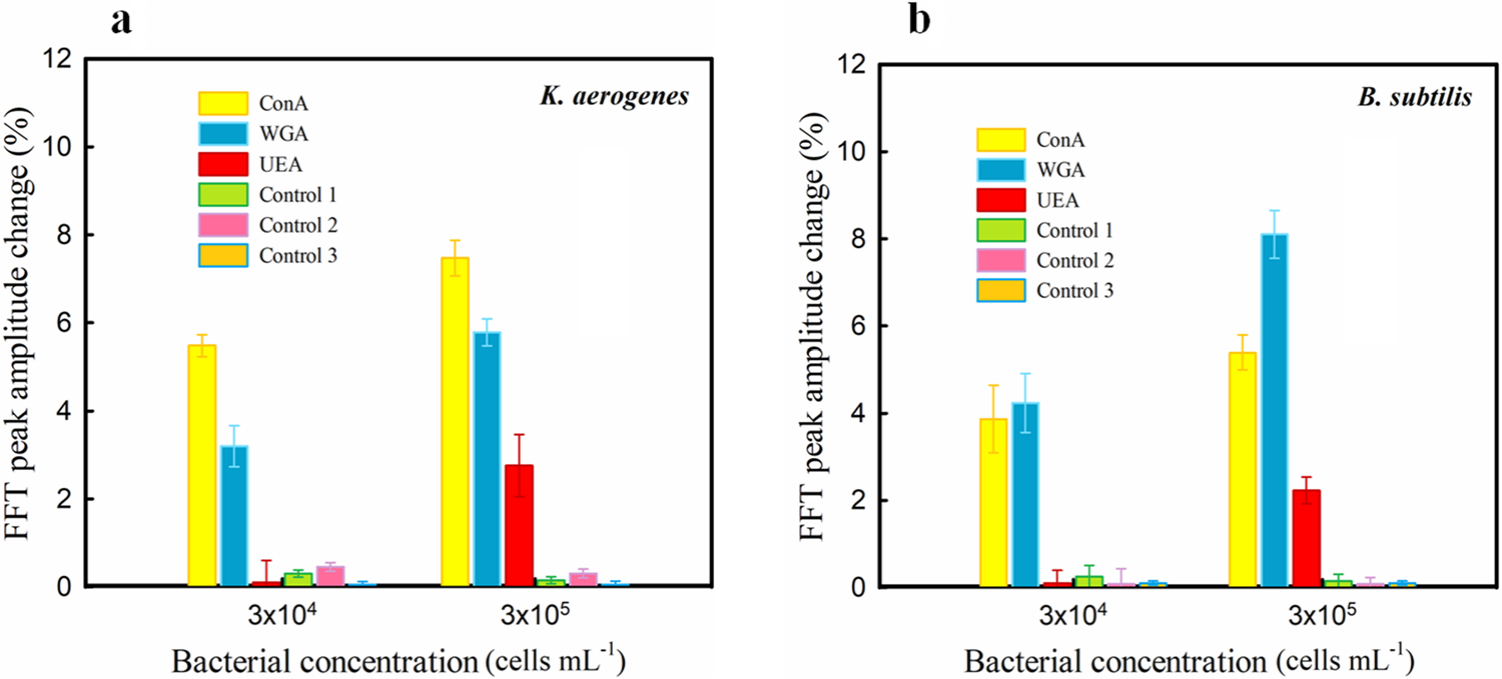
FFT peak amplitude change percentage of PSi modified by three different types of lectin (ConA, WGA, and UEA) at the presence of different concentrations of (**A**) *K. aerogenes* and (**B**) *B. subtilis*. Control tests: fresh PSi as control 1, GA-modified PSi (without lectin) as control 2 and DI water on ConA-modified PSi as control 3.

Regarding the obtained results with *E. coli* and *S. aureus*, it seemed that Gram-negative and Gram-positive bacteria have different interaction patterns based on lectin type. These results encouraged us to test the biosensor’s performance with two other bacteria. We used *Klebsiella aerogenes* and *Bacillus subtilis* strains for more experiments. The obtained results (Fig. 6) showed that the affinity pattern for both recent bacteria follows the same pattern as the previous results for *E. coli* and *S. aureus*. Also, these results reinforced the conjecture that the WGA and ConA have a more interaction affinity for Gram-positive and Gram-negative bacteria, respectively. Of course, to give a definitive opinion, many more bacteria species will need to be tested in the next studies.

Moreover, the biosensor performance analysis with *E. coli* culture medium as a real sample showed that although the response rate (percent of FFT peak amplitude change) has been slightly decreased compared to pure bacterial dilutions (have addressed in manuscript), it still was significantly upper than the control tests.

For example, for 10^–3^ dilution of culture medium, the FFT peak amplitude decrease is 4.1% in contrast to 6.8% for 10^–3^ pure bacterial suspension. Also during spectra recording relatively high ups and downs occurred can be due to the physical adsorption and separation of the culture medium compounds. This result indicated that the designed platform can be efficiently applied even for complex samples and other biosensing purposes.

### Principal component analysis

**Figure 7:**
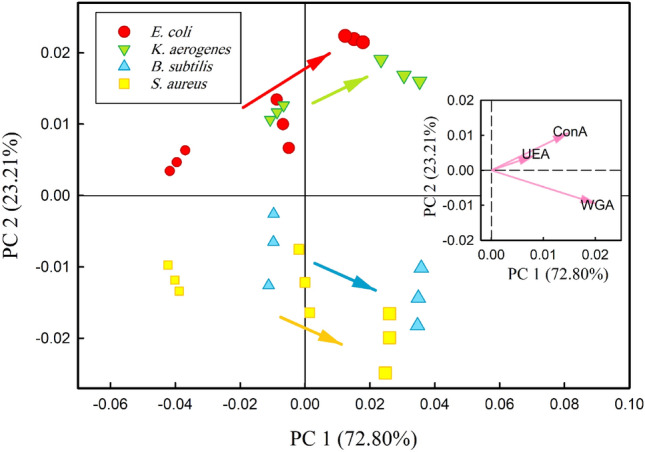
(Main plot) PCA scores plot of the two first PCs. The increase in bacteria concentration is indicated by an arrow direction, (Inset plot) PCA loading plot of the first two principal components.

PCA is a statistically valuable method for discovering relationships between multivariate data. That means the smaller distance between the data indicates a positive correlation and significant similarity between them, and vice versa. In this study, PCA aims to determine the relationship between bacteria strain based on their response profile to different^41,59,60^. The results of the PCA analysis are depicted in Fig. 7. The scores plot (main plot of Fig. 7) shows the projection of the data onto the 2D PCA parameters space. As shown PC1 can explain 72.80% of the total data variance and PC2 can explain 23.21% of the variance. It is obvious from this plot that gram-negative bacteria (*E. coli* and *K. aerogenes*) scatter in a positive PC2 area. However, gram-positive bacteria (*S. aureus* and *B. subtilis*) scatter in the negative PC2 area. Thus, three types of PSi lectin-modified biosensors can discriminate bacterial gram-type effectively without overlap. The loading plot (inset plot in Fig. 7) shows which lectins are significant, and how the three types of PSi lectin-modified biosensors are correlated. In the loading plot, strongly positive correlated variables have two vectors that are very close to each other. However, strongly negative correlated variables have vectors that are out of phase by 180o. Additionally, the magnitude of the vector of each variable also has information. The larger the magnitude of its vector, the stronger impact that variable has on the model^61^. Therefore, the inset plot in Fig. 7 indicates that WGA and ConA have the strongest impact on the responses of our biosensors respectively while UEA has the weakest impact on responses. Besides, ConA and UEA are strongly positive correlated variables. It means that when the response of ConA increases or decreases (by changing the bacteria type or bacteria concentration), the response of UEA tends to change in the same way.

## Conclusion

Advanced and rapid detection methods are mainly based on antibodies or nucleic acids as routine bioreceptor molecules. These two molecules despite sufficient sensitivity and specificity but face challenges due to inherent limitations such as low stability and high cost. On the other hand, the detection methods based on these molecules are mostly time-consuming and require heavy laboratory equipment, qualified personnel, and require some initial knowledge of the target molecule or structure. Hence, in recent years, lectins relying on considerable properties such as low cost, desired stability, acceptable sensitivity, and selectivity has been introduced as an attractive alternative for antibodies and nucleic acids. Therefore in this study, we proposed a lectin conjugated PSi-based biosensor for bacteria detection by the RIFTS technique. Due to the acceptable specificity of lectins, the transducer surface was modified with three different lectins (ConA, WGA, and UEA) as bioreceptors. Evaluation of biosensor performance showed a different response pattern concerning bacteria species and lectin type. So that for *E. coli*, ConA, and *S. aureus*, WGA have the highest binding affinity with a linear rang response from 3 × 10^3^ to 3 × 10^5^ cells mL^−1^ whereas UEA showed the lowest responses to both types of bacteria. Also, a relatively low LOD about 10^3^ cells mL^−1^ was reported for WGA and ConA in their highest binding affinity profile. The further assessments with two extra bacteria species of *K. aerogenes* and *B. subtilis* revealed a similar response pattern based on the lectin type and Gram type of bacteria to the main experiment. Also, the assessment of the obtained data by the PCA test further confirms a significant pattern in the bacteria-lectins interaction based on Gram-type of bacteria. Therefore, given the efficiency and cost-effectiveness of PSi as a transducer and lectin as a bioreceptor, this report can be a promising approach for broad biosensing applications.
